# Microbiomes, diet flexibility, and the spread of a beetle parasite of honey bees

**DOI:** 10.3389/fmicb.2024.1387248

**Published:** 2024-05-31

**Authors:** Qiang Huang, Wensu Han, Francisco Posada-Florez, Jay D. Evans

**Affiliations:** ^1^Honeybee Research Institute, Jiangxi Agricultural University, Nanchang, China; ^2^Department of Integrative Biology, The University of Texas at Austin, Austin, TX, United States; ^3^Environment and Plant Protection Institute, Chinese Academy of Tropical Agricultural Sciences, Haikou, China; ^4^USDA, Beltsville Agricultural Research Center, Bee Research Laboratory, Agricultural Research Service, Beltsville, MD, United States

**Keywords:** invasive pest, symbiosis, beetle, bee, engineered bacteria

## Abstract

Invasive pests may disturb and destructively reformat the local ecosystem. The small hive beetle (SHB), *Aethina tumida*, originated in Africa and has expanded to America, Australia, Europe, and Asia. A key factor facilitating its fast global expansion is its ability to subsist on diverse food inside and outside honey bee colonies. SHBs feed on various plant fruits and exudates in the environment while searching for bee hives. After sneaking into a bee hive, they switch their diet to honey, pollen, and bee larvae. How SHBs survive on such a broad range of food remains unclear. In this study, we simulated the outside and within hive stages by providing banana and hive resources and quantified the SHB associated microbes adjusted by the diet. We found that SHBs fed on bananas were colonized by microbes coding more carbohydrate-active enzymes and a higher alpha diversity than communities from SHBs feeding on hive products or those collected directly from bee hives. SHBs fed on bananas and those collected from the hive showed high symbiont variance, indicated by the beta diversity. Surprisingly, we found the honey bee core symbiont *Snodgrassella alvi* in the guts of SHBs collected in bee hives. To determine the role of *S. alvi* in SHB biology, we inoculated SHBs with a genetically tagged culture of *S. alvi,* showing that this symbiont is a likely transient of SHBs. In contrast, the fungus *Kodamaea ohmeri* is the primary commensal of SHBs. Diet-based microbiome shifts are likely to play a key role in the spread and success of SHBs.

## Introduction

Invasive species are organisms introduced to a new habitat where they are not known to occur. In new habitats, these species thrive and continue to expand their territory (Rohner and Moczek, 2020). Invasive species compete for limited food and shelter resources and parasitize or prey upon local species, causing ecological and economic damage. Invasive species are predicted to have caused 25% of plant extinction and 33% of animal extinction events (Blackburn et al., 2019; Angulo et al., 2022). To which extent species can explore local food resources determines the success of invasions. Species with broad food breadth can have competitive advantages and better pathogenic bacteria tolerance (Barthel et al., 2014; Machovsky-Capuska et al., 2016).

The small hive beetle (*Aethina tumida*, SHB) is a honey bee pest that originated in sub-Saharan Africa. In its native range, honey bees efficiently guard against SHBs, limiting their populations (Neumann et al., 2016; Ouessou Idrissou et al., 2019). Thus, the impact of SHBs on native honey bee colonies is minor. Following international trade lines, SHBs have moved out of Africa and expanded rapidly into novel habitats (Idrissou et al., 2019; Liu et al., 2021). During this expansion, SHBs were first reported in the USA in 1996. SHBs were further dispersed to Australia, Europe, and Asia over two decades (Hood, 2000; Gillespie et al., 2003; Palmeri et al., 2014; Cervancia et al., 2016). SHBs have been introduced in and out of America several times (Neumann et al., 2016). *Kodamaea ohmeri* is a commensal fungus of SHBs, fermenting the honey and eventually sliming the hive, presumably attracting other SHBs (Benda et al., 2008; Amos et al., 2018, 2019). SHBs also carry and disperse bee viruses, causing colony failure (Eyer et al., 2009). SHB infestation has caused substantial damage to the apicultural industry (Hood, 2000; Zhao et al., 2020). One reason for their rapid dispersal is that the SHBs can feed on diverse fruits and saps while searching for bee hives (Stuhl, 2021).

By colonizing the guts or specialized organs, symbionts improve the nutritional yields of flies, bees, aphids, and beetles (Reis et al., 2020; Li et al., 2022; Smith et al., 2022; Luo et al., 2023). In SHBs, neither a symbiont organ nor symbiont shifts in response to nutrition have been reported. Previously, we found distinctive microbes associated with SHB larvae and the co-occurrence of several known bee symbionts (Huang et al., 2019). We hypothesized that the ability to feed on a variety of plant products, including diverse fruits and saps, along with the specialized diet provided by honey bee colonies may require the assistance of gut symbionts. Further, this symbiont community might adjust in response to extreme diets (David et al., 2014). As SHBs may disperse through the banana trade line (Liu et al., 2021), we simulated the dispersal and within hive stages by feeding SHBs with banana (Banana group) and a mixture of bee pollen and honey (Bee_Bread group). We also directly collected SHBs from a honey bee hive (Wild group) to assess natural variation in SHB microbes (Supplementary Figure S1). *Snodgrassella alvi* is a core honey bee gut symbiont. This bacterium colonizes the hindgut, helping lipid metabolism (Quinn et al., 2024). To assess whether honey bee symbionts play a significant role in SHB health, we also used a genetically tagged *S. alvi* and measured the transit of this microbe in the SHB gut.

## Results

### Minor impact of diet on SHBs survival

We first investigated the impact of the banana and bee bread on beetle survival in the lab condition. After 3 weeks of rearing, 30 beetles survived in the Banana group, and 27 survived in the Bee_Bread group (Supplementary Tables S1), hence the variance of the banana and bread on SHBs survival was minor (Pearson’s Chi-squared test, df = 2, *P* = 0.516).

### *De novo* assembly of metagenomes associated with SHBs

**Figure 1:**
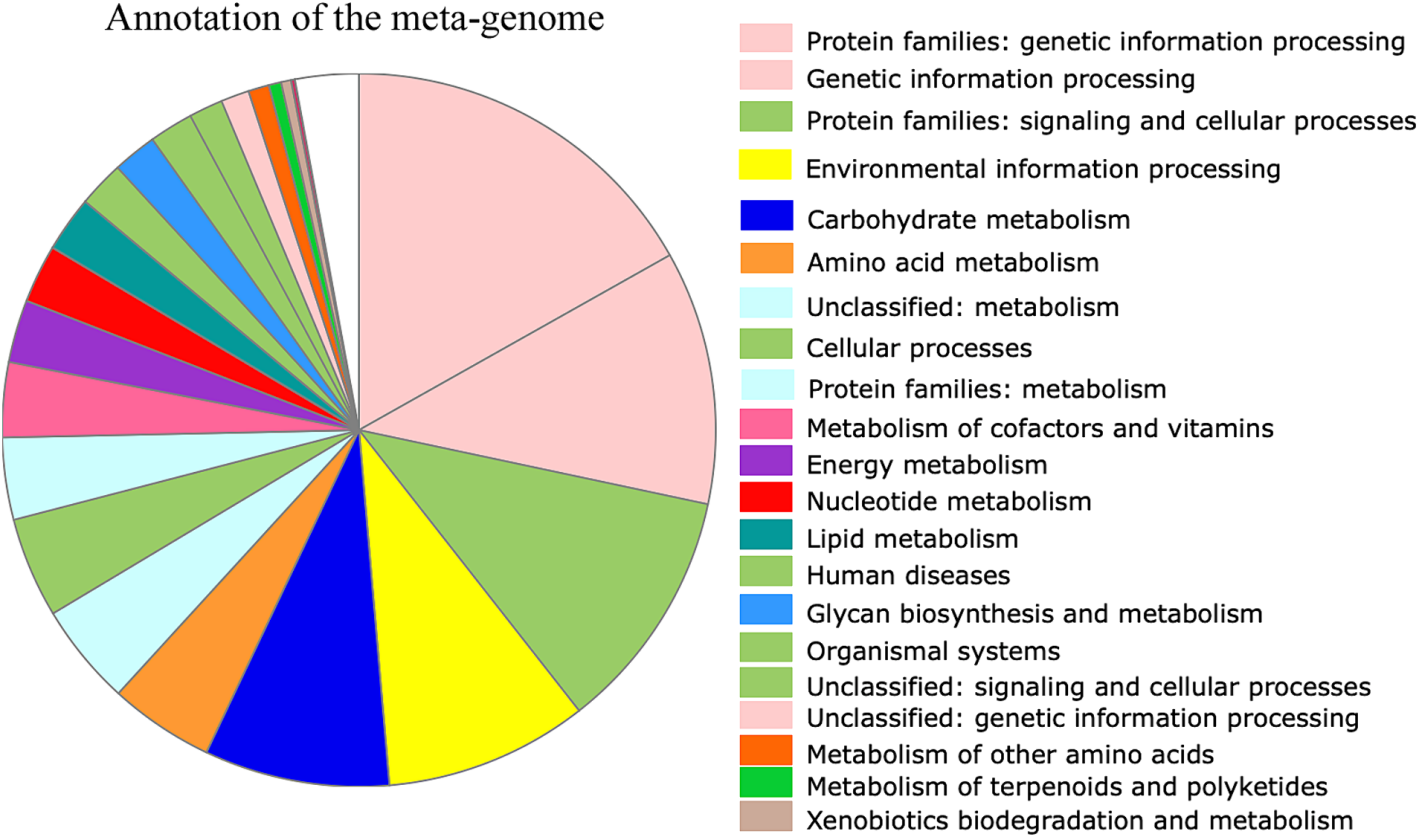
Functional annotation of predicted genes from the assembled metagenomic contigs. Overall, 436,918 genes were predicted, and 200,587 showed functional annotation. Among them, 11,650 genes were involved in carbohydrate metabolism, 6,583 in amino acid metabolism, and 3,471 in lipid metabolism. The color of the functional category was used in global pathway maps and genome maps of KEGG. The functional category on the right was indicated in the pie chart in clockwise order.

As the microbes associated with SHB are largely unknown, we then assembled the microbiome genomes. We randomly selected 10 SHBs in the Banana and Bee_Bread groups and eight SHBs collected in bee hive for metagenomic analyses to quantify the associated gene content and microbes. On average, 5.5 ± 1.3 million reads (150 bp per read) were assigned to microbes in each library, and the ratios of microbe to host reads did not differ significantly between libraries (*t*-test, *P* > 0.05, Supplementary Table S2). We assembled 196,214 metagenome contigs (Supplementary File S2). In those contigs, 436,918 genes were predicted, and 200,587 protein-coding genes retrieved functional annotation in KEGG (Supplementary File S3-S4; Figure 1). The symbionts showed a substantial number of genes involved in nucleotide, energy, carbohydrate, and amino acid metabolism. We also noticed that the symbionts harbor a few xenobiotics biodegradation genes, presumably to degrade toxic compounds (Table 1; Supplementary Table S3).

**Table 1:** The top six enriched pathways modulated by symbiotic genes.

Pathway	KEGG ID	SHB genome	Banana	Bee_Bread	Wild
Purine metabolism	ko00230	270	225	47	131
Pyrimidine metabolism	ko00240	133	146	24	83
Oxidative phosphorylation	ko00190	187	132	26	87
Glycolysis/Glucogeogenesis	ko00010	107	100	14	60
Pyruvate metabolism	ko00620	83	94	17	59
Cysteine and methionine metabolism	ko00270	63	82	16	44

Interestingly, the same pathways were enriched by the diet, even though the number of genes varied. SHB genome indicates the genes in the beetle genomes. Banana indicates symbiotic genes mediated by banana feeding. Bee_Bread indicates symbiotic genes mediated by bee bread feeding. Wild indicates symbiotic genes in wild-collected beetles.

### Banana feeding enhanced metabolic gene number

To quantify the gene copy number, we aligned the reads back to the metagenome. In a pairwise analysis, 15,748 genes were significantly differentially represented between the Banana and Wild groups, 10,678 genes between the Banana and Bee_Bread groups, and 7,994 genes between the Wild and Bee_Bread groups (Table 2; Supplementary Table S4). The Banana group showed a substantially higher number of over-represented genes than the Bee_bread and Wild groups in carbohydrate, lipid, and amino acid metabolism (Chi-squared test, FDR < 0.001). Among the differentially represented genes, 143 carbohydrate-active enzymes (CAZy) were over-represented in the Banana group, compared with 130 in the Wild group and 35 in the Bee_Bread group (Supplementary Table S5). The Banana group showed more CAZy than random among the three treatment groups (Pearson’s Chi-squared test, df = 2, *p* < 0.001).

**Table 2:** The number of over-represented genes in paired groups.

Groups	Number of over-represented genes				Number of over-represented metabolism genes									
	Banana	Bee_Bread	Wild	FDR	Banana			Bee_Bread			Wild			FDR
					C	L	A	C	L	A	C	L	A	
Banana		574	5,616	<0.001				25	7	44	370	165	393	<0.001
Bee_Bread	10,104		5,505	<0.001	781	257	756				366	165	377	<0.001
Wild	10,131	2,489		<0.001	785	256	767	220	63	262				<0.001

The genes were assigned to carbohydrate metabolism (indicated as C), lipid metabolism (indicated as L), and amino acid metabolism (indicated as A). The Banana group showed more over-represented genes than the other two groups in all three metabolism categories when compared with random.

### High microbial diversity in banana feeding SHBs

**Figure 2:**
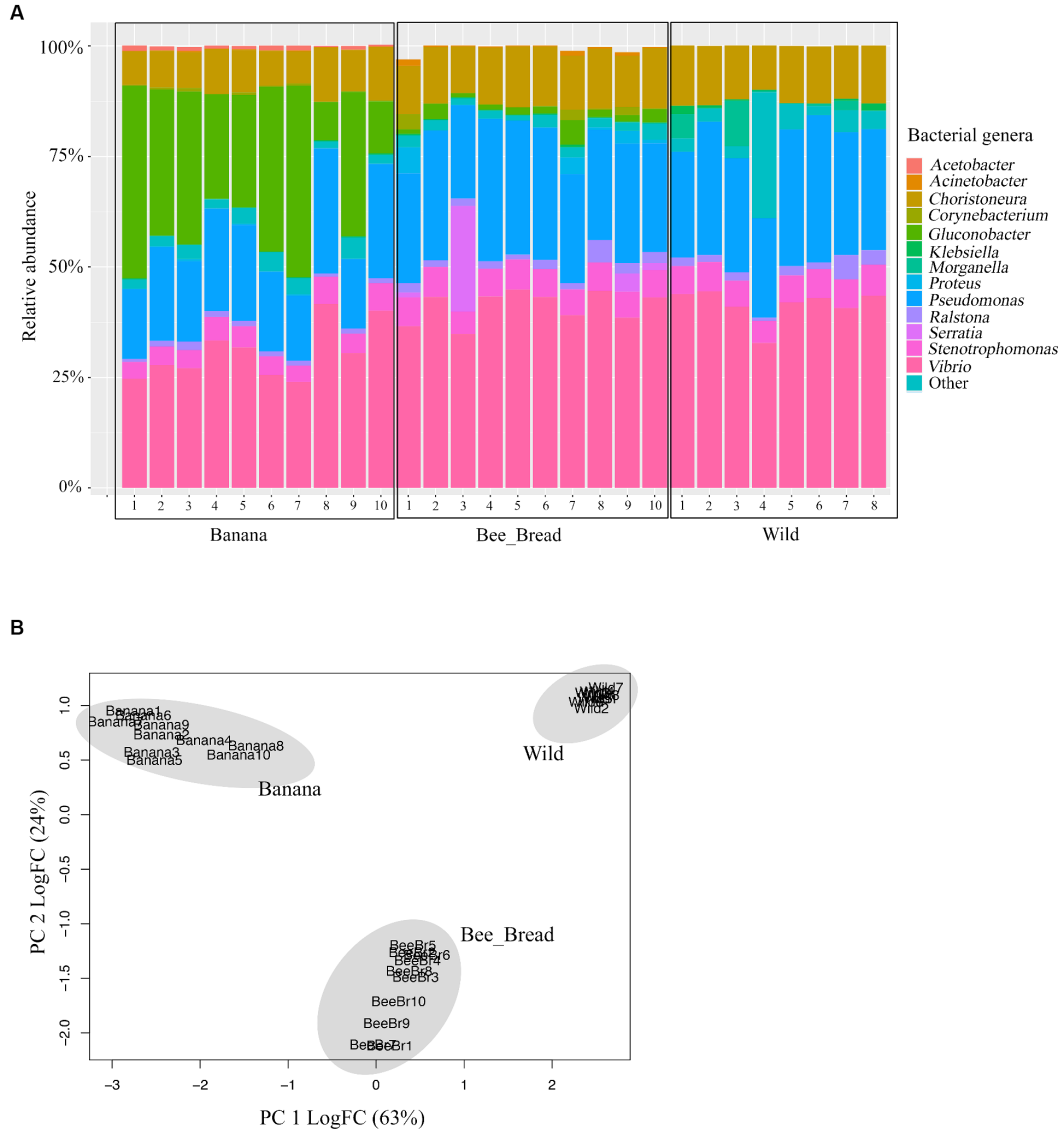
Genome sequencing-based community profiles of the bacteria mediated by the diet in the small hive beetles. **(A)** Relative abundance of the dominant bacterial genera. Banana indicates beetles fed on bananas; Bee_Bread indicates beetles fed on bee bread; Wild indicates beetles collected from bee hives. These bacterial genera were present across all beetle groups at >1% relative abundance. More rare genera (< 1%) were summed up as “Other.” **(B)** PCA plot of the beetles showing the impacts of diet on microbial composition. The microbes were separated by the diet.

**Figure 3:**
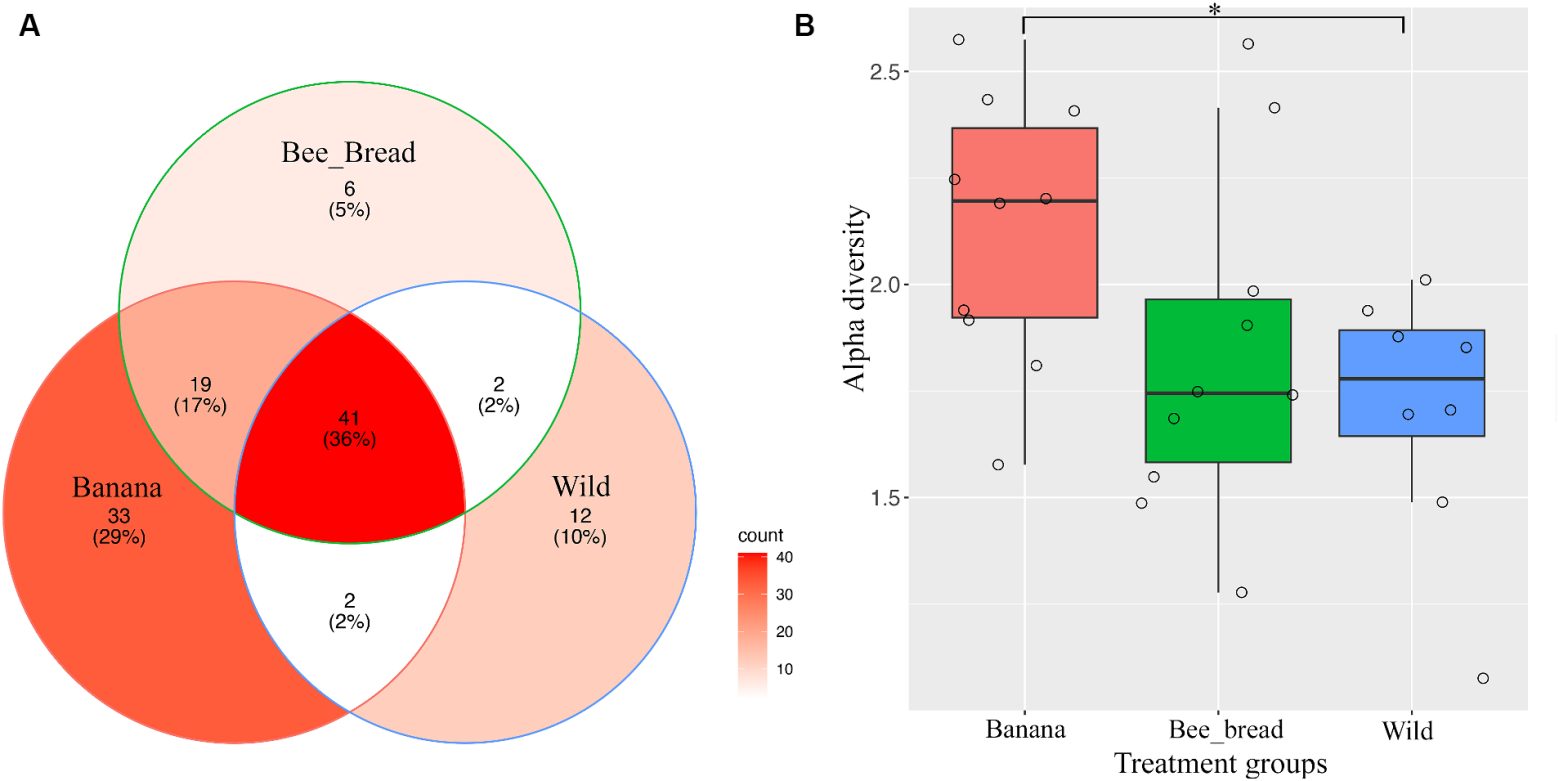
Metagenomic analysis of SHB symbionts. **(A)** Venn diagram of microbes found in the three groups. The banana showed the highest number of microbes and was enriched in acetic-acid bacteria. **(B)** Alpha diversity of the three groups. The banana group showed significantly higher alpha diversity than the Hive group (Wilcoxon rank sum exact test, *p* < 0.05).

To determine microbial diversity, we aligned the microbial reads to the Kraken2 standard database (Supplementary Figure S2-S4). Despite high symbiont diversity overall, 13 bacterial genera dominated the microbial community (> 99% of relative abundance, Figure 2A). The diet separated the microbes and accounted for 87% of the variance (Figure 2B). The Banana group maintained 95 microbial species/strains, followed by 67 in the Bee_Bread group and 56 in the Wild group (Figure 3A). The Banana group showed the highest alpha diversity (2.1 ± 0.31), compared with the Bee_Bread group (1.83 ± 0.40) and the Hive group (1.70 ± 0.30; Kruskal-Wallis test, df = 2, *P* < 0.05, Figure 3B). We also compared the beta diversity between the paired groups. The Wild and Banana group showed the highest beta diversity (0.425), followed by 0.301 between the Wild and Bee_Bread groups. The Banana and Bee_Bread showed the lowest beta diversity of 0.196 (Table 3).

**Table 3:** Beta diversity in the paired groups.

Treatment groups	Bee_Bread	Wild
Banana	0.196	0.425
Bee_Bread		0.301

The highest variance was between the Wild and Banana groups.

We found plant-associated bacteria in the Banana group, such as *Corynebacterium glyciniphilum,* which was the same species initially isolated from the banana for fermentation. The Banana group was significantly enriched in the acetic acid bacteria. Microbes associated with fermentation (*Gluconobacter albidus*, *Mammaliicoccus sciuri*, *Corynebacterium nuruki*) were also highly enriched in the Bee_bread group. We found a few honey bee symbionts in the Wild group, including *Lactococcus lactis* and the core symbiont *Snodgrassella alvi* (Supplementary File S5-S7).

### Honey bee symbiont in SHBs and an antibiotic-resistant commensal fungus

We further tested whether SHB can support the colonization of honey bee gut symbionts. We inoculated newly emerged beetles with a genetically tagged clone of the symbiont *S. alvi* (wkB2:pBTK570). All the beetles survived by the end of the experiment. Tagged symbionts were found in all inoculated beetles, with the average CFU (colony forming unit) of 5,380 at 1 dpi (day post inoculation) on selective plates. Even though the CFU increased to 18,300, the colonization rate dropped to 20% at 3 dpi. When counting the CFU, we observed some microbial colonies morphologically different from *S. alvi* wkB2:pBTK570 in all beetles. To identify the microbial species, we sequenced one such colony, and the ITS2 region aligned with *K. ohmeri* (97% identity, *P* = 7e-97), forming a cluster in the phylogenetic tree (Supplementary Figure S5).

## Discussion

Beetles are the most diverse taxon, making up 40% of all described insect species, including many agricultural pests (Bouchard et al., 2011). From a nutritional point of view, most plant-feeding beetles need more time and enzymes to process large amounts of food because nutrient levels in plants are often low. Symbionts assist beetles in surviving on foods with poor nutritional quality while also helping their hosts cope with toxic plant defenses (Bentz and Six, 2006; Morales-Jiménez et al., 2012; Salem and Kaltenpoth, 2022). In aquatic beetles, symbionts provide essential amino acids and the B vitamin riboflavin for beetle larvae and pectinases to complement host cellulases (Reis et al., 2020). Weevils exhibit convergence in their gut microbial communities when feeding on similar food sources, suggesting that these communities are determined by the environment and host ecology (Berasategui et al., 2016). The gut microbiota help invasive moths feed on new host plant (Zhang et al., 2024). Beetles feed on different plants (generalists) harbor more complex microbes than the specialists (Brunetti et al., 2022). Our study found more diverse microbes in SHBs in the banana group than in the wild group. This suggests that fruit feeding supports more diverse microbes than the protein-rich diet. Alternatively, this high alpha diversity could indicate more transient microbes. Additionally, we found a plant-associated bacterium in the banana group, *C. glyciniphilum,* which was initially isolated from the banana and could temporarily pass through the gut of SHBs (Al-Dilaimi et al., 2015). We also found that SHBs in the banana group were enriched in acetic-acid bacteria, which assist the fermentation of sugar and saps and have established symbioses in bees, flies, and bugs (Crotti et al., 2010).

In the dispersal stage of SHBs, the primary energy source is carbohydrates. Symbiont-mediated carbohydrate-active enzymes (CAZy) facilitate breaking down the major components of plant cell walls, releasing energy sources (Calderón-Cortés et al., 2012; Zheng et al., 2019). In our study, the SHBs fed with banana showed the highest number of CAZy, suggesting that more CAZy genes might be required when providing fruit than the hive resources. In termites, fungus-mediated CAZy assisted the host in decomposing the plant (Poulsen et al., 2014). Symbiont encoding a more dynamic digestive range allows hosts to overcome diet restrictions corresponding to a broader ecological distribution (Salem et al., 2020). In our study, the number of lipid and amino acid metabolism genes was folds higher in the Banana group than in the Bee_Bread and Wild groups. This suggests the symbionts may cooperatively recycle metabolites, as found in social bees (Zheng et al., 2019; Li et al., 2022). When bee hives are located, SHBs sneak in and switch their diet to bee hive resources. Thus, the beetle may switch microbes to adapt to the digestion of hive resources. Future studies quantifying the gene expression and enzymatic activity can explain to what extent the increased gene number reflects functional enhancement.

*K. ohmeri* has been reported to be a commensal fungus in SHBs (Torto et al., 2007; Benda et al., 2008; Amos et al., 2018, 2019). In our study, we found *K. ohmeri* is antibiotic-resistant. It is possible that other SHB-associated antibiotic-resistant bacteria cultivating under different or the same conditions when increasing sequenced colonies. *K. ohmeri* ferments honey and serves as a kairomone to attract other SHBs, a fact used to track and kill SHBs (Stuhl, 2020). The route for beetle progeny to acquire this symbiont remains unclear. We used newly emerged beetles hatched in a new container. Thus, the chance of acquiring fungi from parental feeding or the soil is low. The symbiont might instead be vertically transferred from females. In a previous study, we found the honey bee gut symbiont *S. alvi* in SHBs when feeding the SHBs with bee larvae (Huang et al., 2019). A specialized diet may lead to different gut chemical conditions, creating a gut micro-ecosystem selected for other symbionts (Zmora et al., 2019). For example, *S. alvi* reduces oxygen in the gut, favoring anaerobic microbes and shapes competition (Motta and Moran, 2024). We found this symbiont *S. alvi* again in SHBs metagenome, which is rarely found outside bees. In our data, the colonization rate of *S. alvi* dropped from 100% at 1 dpi to 20% at 3 dpi, even while CFUs increased. This suggests that this bee symbiont cannot consistently colonize SHBs outside the bee hive. In a follow-up study, it will be interesting to reveal the enzymatic activity of bee symbionts in SHBs, to determine if they play a role in SHB fitness while in the hive environment.

## Materials and methods

### SHB rearing, DNA extraction, and Illumina sequencing

We directly collected adult SHBs from collapsed beehives (*Apis cerana*) in a commercial apiary. These beetles were maintained in an incubator (28 ± 1°C temperature and 65% ± 10% humidity), and then we transferred their pupal offspring to a new container until hatching (Neumann et al., 2013). We collected pollen from the honey bee *Apis mellifera* hive entrance. We randomly assigned newly hatched SHBs into two diet groups. SHBs fed on bananas were defined as the Banana group (*N* = 45), and those fed with simulated bee bread (an equal mix of pollen and 50% w/v sugar water to avoid bee hive microbes) comprised the Bee_Bread group (*N* = 45). We then assigned each of the 15 SHBs to a rearing tube. We additionally collected 13 SHBs from a beehive as the wild group because these SHBs were directly from the bee hive without lab feeding (*N* = 13). We refreshed the banana and bee bread daily and collected SHBs after 3 weeks of feeding. We rinsed SHB surfaces with distilled water, then extracted genomic DNA from individual SHBs using MagPure Soil DNA KF Kit (MP Biomedicals, USA). We prepared DNA sequencing libraries using the TruSeq Nano DNA LT Sample Preparation Kit (Illumina, USA). Ten adult SHBs in the Banana group, 10 in the Bee_Bread group, and eight in the Wild group were randomly selected for metagenomic sequencing using the Illumina NovaSeq 6,000 Platform, generating 150 bps paired-end reads.

### *De novo* metagenomic assembly and gene annotation

First, we filtered the sequencing reads using Fastp (Version 0.23.2) with default parameters (Chen et al., 2018). Then we aligned the reads to the small hive beetle genome assembly (GCA_024364675.1) using BWA (Version 0.7.17-r1188) with default parameters and retrieved the unmapped reads using samtools (Version 1.7) and converted the bam to fastq file using bedtools (Version 2.26.0) (Li et al., 2009; Li and Durbin, 2009; Quinlan and Hall, 2010). After that, the unmapped reads were concatenated for all samples to assemble contigs using Megahit (Version 1.2.9) with default parameters (Li et al., 2015), and the contigs were further collapsed using redundans (Version 2020.01.28) (Pryszcz and Gabaldón, 2016). The genes were predicted using MetaGeneMark2 with default parameters.^1^ The protein sequences were queried with the eggNOG-mapper and KEGG database to retrieve the putative function (Cantalapiedra et al., 2021). The code is provided in the Supplementary File S1.

### Gene distribution among microbes associated with diet

The unmapped reads in each beetle were re-aligned to the assembled meta-contigs using BWA (Version 0.7.17-r1188) with default parameters. The number of reads aligned to each gene was quantified using bedtools (Version 2.26.0) (Quinlan and Hall, 2010). The number of aligned reads was normalized to the library size, and the over-represented genes were calculated with edgeR (Quinlan and Hall, 2010). The code is provided in the Supplementary File S1.

### Binning the sequencing reads to microbial species

We performed two steps to bin the sequencing reads to the microbial species. We first aligned the assembled contigs to the most closely related microbes using the BusyBee tool (Laczny et al., 2017). Additionally, we aligned the reads to the Kraken2 (Version 2.1.2) standard database (built on 12/9/2022) (Wood et al., 2019). The number of reads assigned to each microbe was normalized using bracken (Version 2.8) (Lu et al., 2017). The relative abundance of the microbial species was used to calculate alpha (Shannon’s alpha diversity) and beta (Bray–Curtis dissimilarity) diversity using KrakenTools (Lu et al., 2022). The code is provided in the Supplementary File S1.

### Inoculating the bee symbiont to the small hive beetles

As the bee symbionts were constantly reported from SHB metagenomes, we inoculated SHBs with a genetically tagged bee symbiont to validate its colonization. The honey bee symbiont *S. alvi* wkB2:pBTK570 (Addgene accession ID#110615) was previously engineered to be spectinomycin resistant and stored at −80°C (Leonard et al., 2018). This symbiont isolate was activated on Columbia Blood Agar Base (Difco™, 279,220) with 5% sheep blood for 72 h, after which bacterial cells were diluted in 1000 μL PBS and adjusted to OD_600_ = 1. Then, we mixed the homogenized bacterial cells with filter-sterilized 50% sucrose (50%) at a 1:1 ratio. SHBs were fed sucrose with tagged *S. alvi* wkB2:pBTK570 (*N* = 50), then SHBs were rinsed in ethanol and homogenized individually. Homogenates were plated on Columbia Blood Agar Base with 5% sheep blood and spectinomycin (60 μg/mL) to count the Colony Forming Unite (CFU) at 1, 3, 5, and 7 days post-inoculation. Detailed procedures are described in the Supplementary File S1.

### Commensal fungal identification

We observed microbes morphologically distinct from *S. alvi* in all CBA plates. We collected a colony using an inoculating loop to extract DNA using the Qiagen DNeasy Plant Mini kit (cat#69104). The DNA was amplified using fungal ITS primers (FungITS.F 5’GTTAAAAAGCTCGTAGTTG3’; FungITS.R5’CTCTCAATCTGTCAATCCTTATT 3′) in a 30ul reaction consisting of 0.2ul Taq DNA polymerase (Invitrogen, 18038–240), 0.4ul primers, 0.2ul 10uM dNTP mix. The reactions were run with the cycling parameters: 94°C for 3 min., followed by 35 cycles of (94°C 15 s., 54°C 30s., 72°C 1 min.), 72°C for 5 min, and maintained in 4°C. The products were visualized on a 1.75% agarose gel, producing a single band. PCR products were sent for PCR clean-up and Sanger sequencing at Azenta Life Sciences, Rockville, Maryland. Sequences were searched in the NCBI Blastn MegaBlast. An additional nine sequences of fungal species were downloaded from NCBI and aligned with MUSCLE (Version 5.1) with default parameters. The tree was built with MrBayes (Version 3.2.7) and viewed with FigTree (Version 1.4.4). Detailed procedures are described in the Supplementary File S1.

### Statistics

We performed all statistics with R (Version 4.2.2) in RStudio (Version 2022.12.0) (R Core Team, 2013; RStudio Team, 2020). We compared surviving SHBs using Pearson’s Chi-squared test. The number of differentially enriched genes among the paired comparisons was analyzed with Pearson’s Chi-squared test and viewed with the VennDiagram package (Gao et al., 2021). The number of genes in metabolic categories was compared with random using a Chi-squared test, adjusted with the false discovery rate (FDR). The alpha diversity was first analyzed using the Kruskal-Wallis rank sum test, followed by pairwise comparisons using the Wilcoxon rank sum exact test, adjusted with FDR.

## Data availability statement

The original contributions presented in the study are publicly available. This data can be found at the National Center for Biotechnology Information (NCBI) using accession number PRJNA953927.

## Ethics statement

The study involves small hive beetles, which are neither endangered nor a protected insect. No specific permit is required for the described study.

## Author contributions

QH: Formal analysis, Funding acquisition, Writing – original draft. WH: Resources, Writing – original draft. FP-F: Resources, Writing – original draft. JE: Funding acquisition, Writing – review & editing.

## Footnotes

^1^ https://github.com/gatech-genemark/MetaGeneMark-2
