# Supplementary material for: Microbiomes, diet flexibility, and the spread of a beetle parasite of honey bees
Source: Front Microbiol. 2024 May 31;15:1387248. doi: 10.3389/fmicb.2024.1387248 (PMC11176428; doi:10.3389/fmicb.2024.1387248)
Supplement: Supplementary file 1 [file Data_Sheet_1.pdf]

Supporting Information for

Microbiomes, diet flexibility, and the spread of a beetle parasite of honey bees

Qiang Huang[1,2*], Wensu Han[3], Francisco Posada-Florez[4], Jay D. Evans[4]

[1] Honeybee Research Institute, Jiangxi Agricultural University, Zhimin Ave 1101, 330045, Nanchang, China.

[2] Department of Integrative Biology, The University of Texas at Austin, Austin, TX, 78712, USA.

[3] Environment and Plant Protection Institute, Chinese Academy of Tropical Agricultural Sciences, Haikou, China.

[4] USDA, Agricultural Research Service, Beltsville Agricultural Research Center, Bee Research Laboratory, 10300 Baltimore Avenue, 20705, Beltsville, MD, USA.

Material and Methods

De novo metagenomic assembly and gene annotation

```
# 1. Filtering sequencing reads
Fastp -i SHB1.1.fq -I SHB1.2.fq -o SHB1.1.filtered.fq -O SHB1.2.filtered.fq

 # 2. remove the reads from the small hive beetle genome
bwa mem -t 16 SHB_reference_genome SHB1.1.filtered.fq SHB1.2.filtered.fq > SHB1.sam
samtools view -S -b SHB1.sam > SHB1.bam
samtools sort SHB1.bam > SHB1.sorted.bam
samtools view -b -f 4 SHB1.sorted.bam > SHB1.unmapped.bam
bedtools bamtofastq -i SHB1.unmapped.bam -fq SHB1_microbe.1.fq -fq2 SHB1_microbe.2.fq

#3 concentrate microbe reads for all 28 SHB samples
cat SHB1_microbe.1.fa  ... SHB28_microbe.1.fa   > all.unmapped1.fq
cat SHB1_microbe.2.fa  ... SHB28_microbe.2.fa   > all.unmapped2.fq

#4 assemble the metagenome contigs
megahit -1 all.unmapped1.fq -2 all.unmapped2.fq -o megahit_output

#5 collapse the redundant contigs
```

```
redundans.py -t 16 all.unmapped1.fq all.unmapped2.fq -f final.contigs.fa -o redundans
```

```
#6 gene annotation
Perl run_mgm.pl meta.contigs.fa --nt cds.fa --aa protein.fa --format gff
```

Bin reads to the microbial species using Kraken2

```
kraken2 -db K2_database --threads 8 --report SHB1_kraken_report --report-minimizer-data --minimum-hit-groups 3 paired SHB1_microbe.1.fq SHB1_microbe.2.fq > SHB1.kraken.output
```

Two output files
SHB1_report.txt
SHB1.kraken2.output

```
bracken -d K2_database -i SHB1_kraken_report -l S -o SHB1.bracken.output -t 10 -w SHB1.bracken.report
```

Two output files
SHB1.kraken2.bracken.output
SHB1_report_bracken_species.txt

Honey bee gut symbiont engineering

The gut symbiont *Snodgrassella alvi*, strain wkB2 (NCBI: TXID 1196094), was previously isolated and cultivated on Columbia Blood Agar Base (Difco^{TM}, 279220) with 5% sheep blood. To prepare electrocompetent cells, a single colony of *S. alvi* wkB2 was added to 50mL Columbia Broth (Difco^{TM}, 294420), incubating at 35°C with 5% CO_2 for 72 hours. Then the liquid media was centrifuged at 7,000 rpm for 10 min to harvest the pellet. The pellet was washed with autoclaved ultra-pure water three times, and the pellet was re-suspended in 1 mL of autoclaved 10% glycerol. pBTK 570 (no expression of dsRNA, Addgene accession ID#110615) was introduced into *S. alvi* wkB2.

Table S1 The number of survived beetles in each food type. In the Banana and Bee_Bread groups, 45 beetles were assigned to three rearing tubes. After three weeks of rearing, 30 and 27 beetles survived in the Banana and Bee_Bread groups. The number of survived beetles was not significantly different between the Banana and Bee_Bread groups.

Food type	Tested beetles	Survived beetles	Sequenced beetles	Statistics
Banana	45	30	10	Pearson's Chi-squared test, df=2, *P*=0.516
Bee bread	45	27	10	

Table S2 Alignment statistics of the reads to the Small Hive Beetle (SHB) genome assembly. In total, 28 beetles were individually sequenced. Approximately 2.06% of total reads (5.5 million reads) cannot be aligned to the beetle genome. Those unmapped reads were used to proceed with the metagenomic analysis.

Samples	Total	mapped to SHB	unmapped to SHB
Banana-1	221,997,194	217,383,400	4,613,794
Banana-2	284,888,195	278,622,256	6,265,939
Banana-3	388,169,594	380,876,484	7,293,110
Banana-4	421,007,904	412,444,691	8,563,213
Banana-5	223,633,306	218,149,502	5,483,804
Banana-6	232,833,812	227,505,353	5,328,459
Banana-7	239,248,413	233,071,375	6,177,038
Banana-8	358,814,940	349,204,606	9,610,334
Banana-9	229,450,421	224,123,251	5,327,170
Banana-10	227,763,701	222,576,145	5,187,556
Bee_Br-1	295,544,126	289,579,893	5,964,233
Bee_Br-2	287,579,500	282,726,602	4,852,898
Bee_Br-3	279,774,064	274,274,564	5,499,500
Bee_Br-4	246,226,073	240,386,742	5,839,331
Bee_Br-5	231,071,267	225,355,158	5,716,109
Bee_Br-6	230,984,253	225,470,285	5,513,968
Bee_Br-7	239,248,413	233,071,375	6,177,038
Bee_Br-8	246,524,253	242,296,425	4,227,828
Bee_Br-9	247,380,120	243,300,057	4,080,063
Bee_Br-10	245,478,883	240,714,912	4,763,971
Wild-1	256,774,431	252,904,159	3,870,272
Wild-2	256,854,778	250,471,761	6,383,017
Wild-3	227,853,402	224,127,588	3,725,814
Wild-4	206,690,221	201,768,410	4,921,811
Wild-5	232,605,332	228,321,842	4,283,490
Wild-6	222,332,984	218,363,590	3,969,394
Wild-7	435,069,678	429,582,238	5,487,440
Wild-8	413,377,351	408,100,347	5,277,004

Table S3 The number of genes assigned to the functional pathways. The symbiont-mediated genes enhanced overall metabolic capacity of the SHBs.

		SHB genome	Banana	Beebread	Wild
00230 Purine metabolism	ko00230	270	225	47	131
00240 Pyrimidine metabolism	ko00240	133	146	24	83
00190 Oxidative phosphorylation	ko00190	178	132	26	87
00010 Glycolysis / Gluconeogenesis	ko00010	107	100	14	60
00620 Pyruvate metabolism	ko00620	83	94	17	59
00270 Cysteine and methionine metabolism	ko00270	63	82	16	44
00250 Alanine, aspartate and glutamate metabolism	ko00250	49	70	11	42
00260 Glycine, serine and threonine metabolism	ko00260	57	69	16	40
00630 Glyoxylate and dicarboxylate metabolism	ko00630	61	69	16	40
00020 Citrate cycle (TCA cycle)	ko00020	85	68	13	44
00480 Glutathione metabolism	ko00480	108	67	8	30
00520 Amino sugar and nucleotide sugar metabolism	ko00520	80	66	11	39
00564 Glycerophospholipid metabolism	ko00564	211	66	15	38
00500 Starch and sucrose metabolism	ko00500	76	64	10	39
00680 Methane metabolism	ko00680	33	63	8	34
00330 Arginine and proline metabolism	ko00330	65	61	11	35
00030 Pentose phosphate pathway	ko00030	63	61	6	29
00310 Lysine degradation	ko00310	94	51	6	22
00513 Various types of N-glycan biosynthesis	ko00513	66	49	7	34
00561 Glycerolipid metabolism	ko00561	125	49	11	31
00562 Inositol phosphate metabolism	ko00562	103	48	9	29
00720 Carbon fixation pathways in prokaryotes	ko00720	24	48	5	25
00051 Fructose and mannose metabolism	ko00051	56	47	4	26
00510 N-Glycan biosynthesis	ko00510	53	46	6	35
00360 Phenylalanine metabolism	ko00360	37	42	8	21
00400 Phenylalanine, tyrosine and tryptophan biosynthesis	ko00400	7	41	7	19
00220 Arginine biosynthesis	ko00220	24	40	7	22
00710 Carbon fixation in photosynthetic organisms	ko00710	48	40	7	27
00640 Propanoate metabolism	ko00640	46	39	5	22
00860 Porphyrin metabolism	ko00860	97	38	6	22
00350 Tyrosine metabolism	ko00350	49	37	7	21
00340 Histidine metabolism	ko00340	25	37	8	17
00760 Nicotinate and nicotinamide metabolism	ko00760	55	37	8	18
00380 Tryptophan metabolism	ko00380	67	36	7	25
00071 Fatty acid degradation	ko00071	55	36	4	20

00900 Terpenoid backbone biosynthesis	ko00900	49	35	11	27
00670 One carbon pool by folate	ko00670	24	33	2	17
00920 Sulfur metabolism	ko00920	20	32	4	24
00061 Fatty acid biosynthesis	ko00061	23	31	3	15
00563 Glycosylphosphatidylinositol (GPI)-anchor biosynthesis	ko00563	27	30	10	28
00290 Valine, leucine and isoleucine biosynthesis	ko00290	3	29	3	16
00770 Pantothenate and CoA biosynthesis	ko00770	20	29	4	17
00410 beta-Alanine metabolism	ko00410	35	27	3	18
00300 Lysine biosynthesis	ko00300	1	26	5	16
00280 Valine, leucine and isoleucine degradation	ko00280	60	26	3	15
00052 Galactose metabolism	ko00052	76	26	6	17
00650 Butanoate metabolism	ko00650	24	25	6	15
00450 Selenocompound metabolism	ko00450	15	24	7	11
00982 Drug metabolism - cytochrome P450	ko00982	142	24	3	12
00100 Steroid biosynthesis	ko00100	70	23	5	20
00460 Cyanoamino acid metabolism	ko00460	25	22	2	10
00980 Metabolism of xenobiotics by cytochrome P450	ko00980	145	22	1	11
00730 Thiamine metabolism	ko00730	23	21	4	13
00040 Pentose and glucuronate interconversions	ko00040	130	20	5	14
00910 Nitrogen metabolism	ko00910	18	20	4	10
00514 Other types of O-glycan biosynthesis	ko00514	21	20	4	17
01040 Biosynthesis of unsaturated fatty acids	ko01040	33	20	6	15
00960 Tropane, piperidine and pyridine alkaloid biosynthesis	ko00960	5	19	3	9
00790 Folate biosynthesis	ko00790	60	19	1	9
00130 Ubiquinone and other terpenoid-quinone biosynthesis	ko00130	22	16	5	9
00780 Biotin metabolism	ko00780	9	16	3	9
00430 Taurine and hypotaurine metabolism	ko00430	14	16	2	11
00625 Chloroalkane and chloroalkene degradation	ko00625	9	16	1	9
00053 Ascorbate and aldarate metabolism	ko00053	100	15	1	8
00600 Sphingolipid metabolism	ko00600	87	15	4	13
00750 Vitamin B6 metabolism	ko00750	15	15	3	8
00950 Isoquinoline alkaloid biosynthesis	ko00950	26	14	5	9
00521 Streptomycin biosynthesis	ko00521	14	13	3	8
00062 Fatty acid elongation	ko00062	32	13	2	9
00565 Ether lipid metabolism	ko00565	76	13	2	7
00550 Peptidoglycan biosynthesis	ko00550	0	12	0	6
00740 Riboflavin metabolism	ko00740	26	12	2	7
00940 Phenylpropanoid biosynthesis	ko00940	20	11	4	7

00511 Other glycan degradation	ko00511	63	11	3	7
00830 Retinol metabolism	ko00830	111	11	1	6
00627 Aminobenzoate degradation	ko00627	5	11	2	8
00401 Novobiocin biosynthesis	ko00401	1	9	1	2
00590 Arachidonic acid metabolism	ko00590	63	9	3	6
00660 C5-Branched dibasic acid metabolism	ko00660	0	8	1	3
00515 Mannose type O-glycan biosynthesis	ko00515	26	8	1	7
00540 Lipopolysaccharide biosynthesis	ko00540	0	8	1	1
00981 Insect hormone biosynthesis	ko00981	110	8	0	5
00930 Caprolactam degradation	ko00930	39	8	2	6
00626 Naphthalene degradation	ko00626	1	8	0	4
00362 Benzoate degradation	ko00362	6	8	2	3
00261 Monobactam biosynthesis	ko00261	2	7	1	5
00140 Steroid hormone biosynthesis	ko00140	95	7	2	5
00909 Sesquiterpenoid and triterpenoid biosynthesis	ko00909	0	7	1	4
00903 Limonene degradation	ko00903	8	7	0	4
00524 Neomycin, kanamycin and gentamicin biosynthesis	ko00524	7	6	1	4
00195 Photosynthesis	ko00195	0	6	0	0
00785 Lipoic acid metabolism	ko00785	4	6	1	4
00531 Glycosaminoglycan degradation	ko00531	25	5	0	0
00592 alpha-Linolenic acid metabolism	ko00592	31	5	3	5
00908 Zeatin biosynthesis	ko00908	2	5	0	2
00643 Styrene degradation	ko00643	3	5	1	3
00254 Aflatoxin biosynthesis	ko00254	3	4	1	4
00311 Penicillin and cephalosporin biosynthesis	ko00311	3	4	1	2
00333 Prodigiosin biosynthesis	ko00333	1	4	0	2
00361 Chlorocyclohexane and chlorobenzene degradation	ko00361	0	4	1	2
00332 Carbapenem biosynthesis	ko00332	0	3	1	2
00405 Phenazine biosynthesis	ko00405	0	3	0	1
00603 Glycosphingolipid biosynthesis - globo and isoglobo series	ko00603	22	3	0	0
00604 Glycosphingolipid biosynthesis - ganglio series	ko00604	8	3	0	0
01051 Biosynthesis of ansamycins	ko01051	2	3	0	2
00364 Fluorobenzoate degradation	ko00364	0	3	0	1
00623 Toluene degradation	ko00623	0	3	0	1
00120 Primary bile acid biosynthesis	ko00120	12	2	0	2
00440 Phosphonate and phosphinate metabolism	ko00440	11	2	1	2
00791 Atrazine degradation	ko00791	0	2	0	2

00945 Stilbenoid, diarylheptanoid and gingerol biosynthesis	ko00945	0	1	1	1
00232 Caffeine metabolism	ko00232	19	1	1	1
00941 Flavonoid biosynthesis	ko00941	0	1	1	1
00966 Glucosinolate biosynthesis	ko00966	0	1	0	0
00512 Mucin type O-glycan biosynthesis	ko00512	21	1	0	0
00532 Glycosaminoglycan biosynthesis - chondroitin sulfate / dermatan sulfate	ko00532	24	1	1	1
00534 Glycosaminoglycan biosynthesis - heparan sulfate / heparin	ko00534	24	1	1	1
00591 Linoleic acid metabolism	ko00591	29	1	0	1
00906 Carotenoid biosynthesis	ko00906	0	1	0	0
01053 Biosynthesis of siderophore group nonribosomal peptides	ko01053	0	1	0	0
00642 Ethylbenzene degradation	ko00642	0	1	1	1
00633 Nitrotoluene degradation	ko00633	0	1	0	0
00965 Betalain biosynthesis	ko00965	14	0	0	0
00901 Indole alkaloid biosynthesis	ko00901	10	0	0	0
00944 Flavone and flavonol biosynthesis	ko00944	2	0	0	0
00942 Anthocyanin biosynthesis	ko00942	0	0	0	0
00943 Isoflavonoid biosynthesis	ko00943	0	0	0	0
00946 Degradation of flavonoids	ko00946	0	0	0	0
00403 Indole diterpene alkaloid biosynthesis	ko00403	0	0	0	0
00996 Biosynthesis of various alkaloids	ko00996	0	0	0	0
00402 Benzoxazinoid biosynthesis	ko00402	0	0	0	0
00331 Clavulanic acid biosynthesis	ko00331	0	0	0	0
00525 Acarbose and validamycin biosynthesis	ko00525	0	0	0	0
00404 Staurosporine biosynthesis	ko00404	0	0	0	0
00998 Biosynthesis of various antibiotics	ko00998	0	0	0	0
00999 Biosynthesis of various plant secondary metabolites	ko00999	0	0	0	0
00997 Biosynthesis of various other secondary metabolites	ko00997	0	0	0	0
00196 Photosynthesis - antenna proteins	ko00196	0	0	0	0
00601 Glycosphingolipid biosynthesis - lacto and neolacto series	ko00601	32	0	0	0
00533 Glycosaminoglycan biosynthesis - keratan sulfate	ko00533	3	0	0	0
00542 O-Antigen repeat unit biosynthesis	ko00542	0	0	0	0
00541 O-Antigen nucleotide sugar biosynthesis	ko00541	0	0	0	0
00552 Teichoic acid biosynthesis	ko00552	0	0	0	0
00571 Lipoarabinomannan (LAM) biosynthesis	ko00571	0	0	0	0
00572 Arabinogalactan biosynthesis - Mycobacterium	ko00572	0	0	0	0
00543 Exopolysaccharide biosynthesis	ko00543	0	0	0	0

00073 Cutin, suberine and wax biosynthesis	ko00073	35	0	0	0
00121 Secondary bile acid biosynthesis	ko00121	0	0	0	0
00470 D-Amino acid metabolism	ko00470	0	0	0	0
00902 Monoterpenoid biosynthesis	ko00902	0	0	0	0
00904 Diterpenoid biosynthesis	ko00904	0	0	0	0
00905 Brassinosteroid biosynthesis	ko00905	0	0	0	0
00907 Pinene, camphor and geraniol degradation	ko00907	0	0	0	0
01052 Type I polyketide structures	ko01052	0	0	0	0
00522 Biosynthesis of 12-, 14- and 16-membered macrolides	ko00522	0	0	0	0
01059 Biosynthesis of enediyne antibiotics	ko01059	0	0	0	0
01056 Biosynthesis of type II polyketide backbone	ko01056	0	0	0	0
01057 Biosynthesis of type II polyketide products	ko01057	0	0	0	0
00253 Tetracycline biosynthesis	ko00253	0	0	0	0
00523 Polyketide sugar unit biosynthesis	ko00523	0	0	0	0
01054 Nonribosomal peptide structures	ko01054	0	0	0	0
01055 Biosynthesis of vancomycin group antibiotics	ko01055	0	0	0	0
00622 Xylene degradation	ko00622	0	0	0	0
00363 Bisphenol degradation	ko00363	0	0	0	0
00621 Dioxin degradation	ko00621	0	0	0	0
00624 Polycyclic aromatic hydrocarbon degradation	ko00624	0	0	0	0
00365 Furfural degradation	ko00365	0	0	0	0
00984 Steroid degradation	ko00984	0	0	0	0

Table S4 The number of significantly differentially represented genes in the three paired comparisons. The largest difference was observed between the Banana and Hive groups, and the least was observed between the Bee_Bread and Hive groups.

Groups	Banana	Hive
Bee_Bread	10,678	7,994
Hive	15,747	NA

Table S5 Carbohydrate-active enzymes (CAZy) identified in the Banana and Wild groups.

**Group**	**Gene**	**PFAMs**	**CAZy**
Banana	k141_10761_1	COesterase	CE10
Banana	k141_316204_14	3D,MltA	GH102
Banana	k141_344616_2	Fn3-like,Glyco_hydro_3,Glyco_hydro_3_C,PA14	GH3

Banana	k141_396860_1	Trehalase,Trehalase_Ca-bi	GH37
Banana	k141_172278_3	Big_2,CBM_35,Glyco_hydro_66,Inhibitor_I9,PA,Peptidase_S8,SLH	GH66
Banana	k141_219979_4	Glyco_hydro_68	GH68
Banana	k141_324551_2	Glyco_hydro_76	GH76
Banana	k141_359949_1	Glyco_transf_15	GT15
Banana	k141_370052_4	LpxB	GT19
Banana	k141_108768_1	Chitin_synth_1,Chitin_synth_1N	GT2
Banana	k141_401066_7	Glycos_transf_2	GT2
Banana	k141_37992_5	Glycos_transf_2,GtrA	GT2
Banana	k141_429319_12	CN_hydrolase	GT2
Banana	k141_174737_1	Glyco_transf_8,UDP-g_GGTase	GT24
Banana	k141_198491_2	DAO	GT58
Banana	k141_121391_1	Mannosyl_trans3	GT71
Banana	k141_384827_3	AftA_C,AftA_N	GT85
Wild	k141_381255_1	Alpha-amylase,Alpha-amylase_C,CBM_48	CBM48,GH13
Wild	k141_439896_2	Glyco_hydro_15	GH15
Wild	k141_54549_1	SKN1	GH16
Wild	k141_346949_1	CBM_1,CBM_19,Glyco_hydro_18	GH18
Wild	k141_30431_1	Glycogen_syn	GT3
Wild	k141_336027_3	Gly_transf_sug	GT32
Wild	k141_204936_1	Gly_transf_sug	GT32
Wild	k141_286250_6	Glyco_trans_1_4,Glyco_trans_4_4,Glyco_transf_4,Glycos_transf_1	GT33
Wild	k141_232160_1	MIR,PMT	GT39
Wild	k141_182377_1	MIR,PMT,PMT_4TMC	GT39
Wild	k141_350083_1	Mannosyl_trans3	GT71
Wild	k141_47664_1	Mannosyl_trans3	GT71

Table S6 primer and PCR sequences for the single colony isolate.

Forward primer	Reverse primer	PCR product
GTTAAAAAGCTCGTAGTTG	CTCTCAATCTGTCAATCCTTATT	CTACTGACCGGCACGCACCTTTCCTTCTGGTTTTCGGACCAGGACG TTTACTTTGAAAAAATTAGAGTGTTCAAAGCAGGCATATTGCTCGAAT ATATTAGCATGGAATAAGGGAATAGGACAATGGTTCTATTTCGTTGG TTTTCAGTACCATTGTAATGATTAATAGGGACGGACGGGGGCNNCAG TANTCCGTAATCNGANGTGAAATT

	**Treatment**	**Analysis**
Experiment 1	Bee bread feeding<br>Banana feeding<br>Wild beetle collection	survival rate<br>microbial binning<br>gene content analysis
Experiment 2	symbiont inoculation	CFU counting<br>microbial indentification

Figure S1 Experimental schemes for this study. In experiment 1, we fed beetles with different diets to assess its impacts on the survival and the associated microbial, as well as gene content after de novo assembly. In experiment 2, we inoculated beetles with genetically tagged symbiont to quantify its colonization.

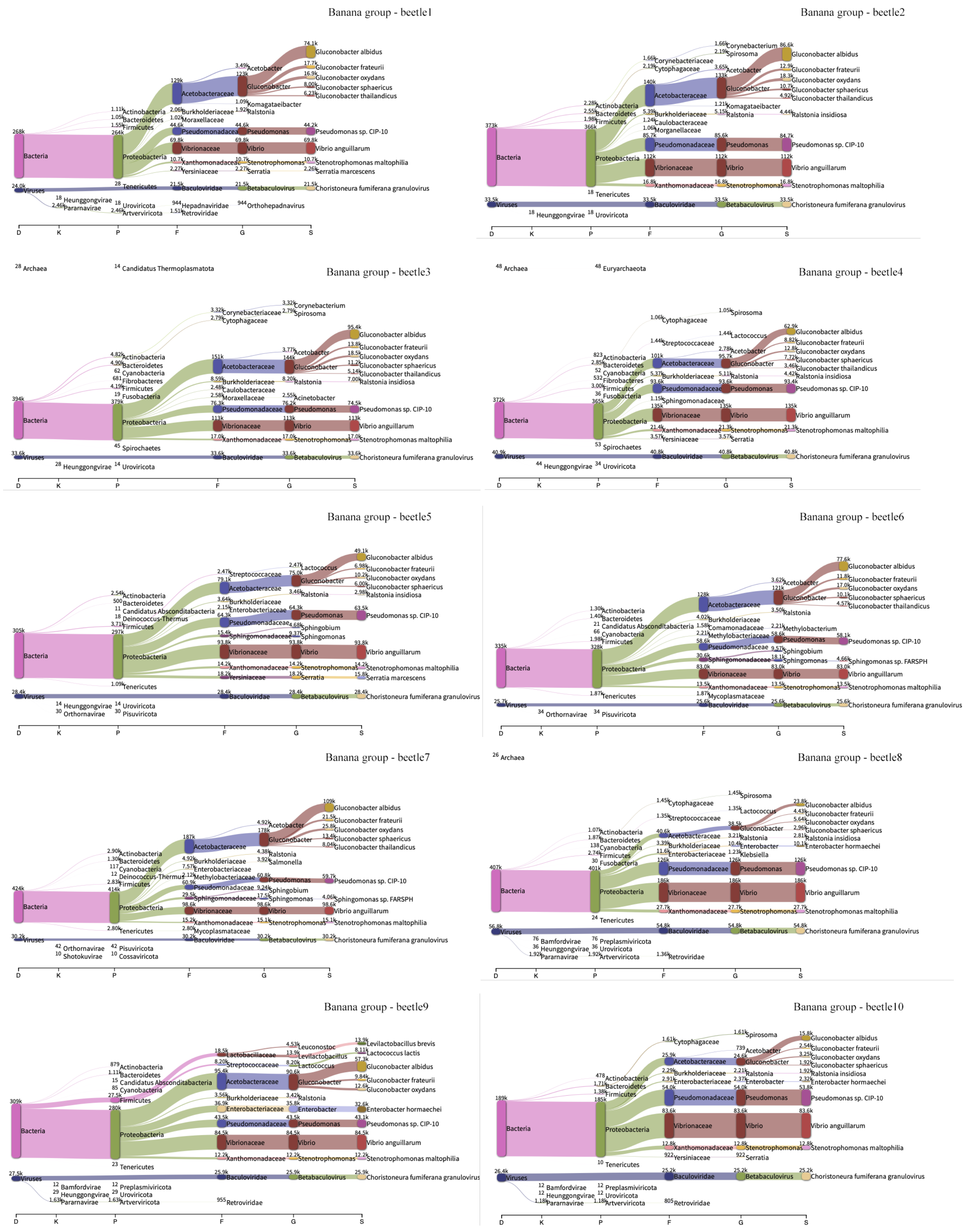

Figure S2 Pavian output for hierarchical visualization in the Banana group.

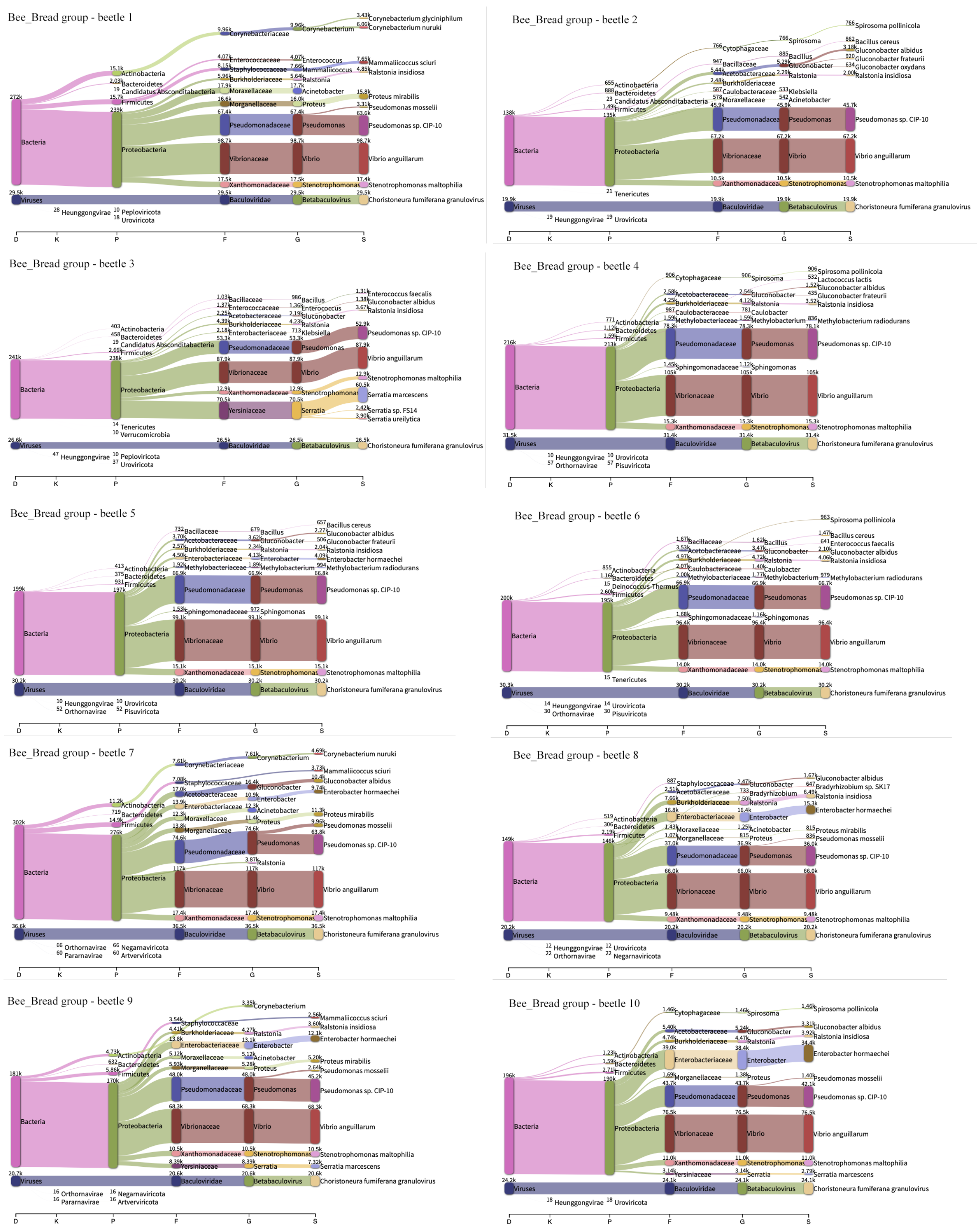

Figure S3 Pavian output for hierarchical visualization in the Bee_Bread group

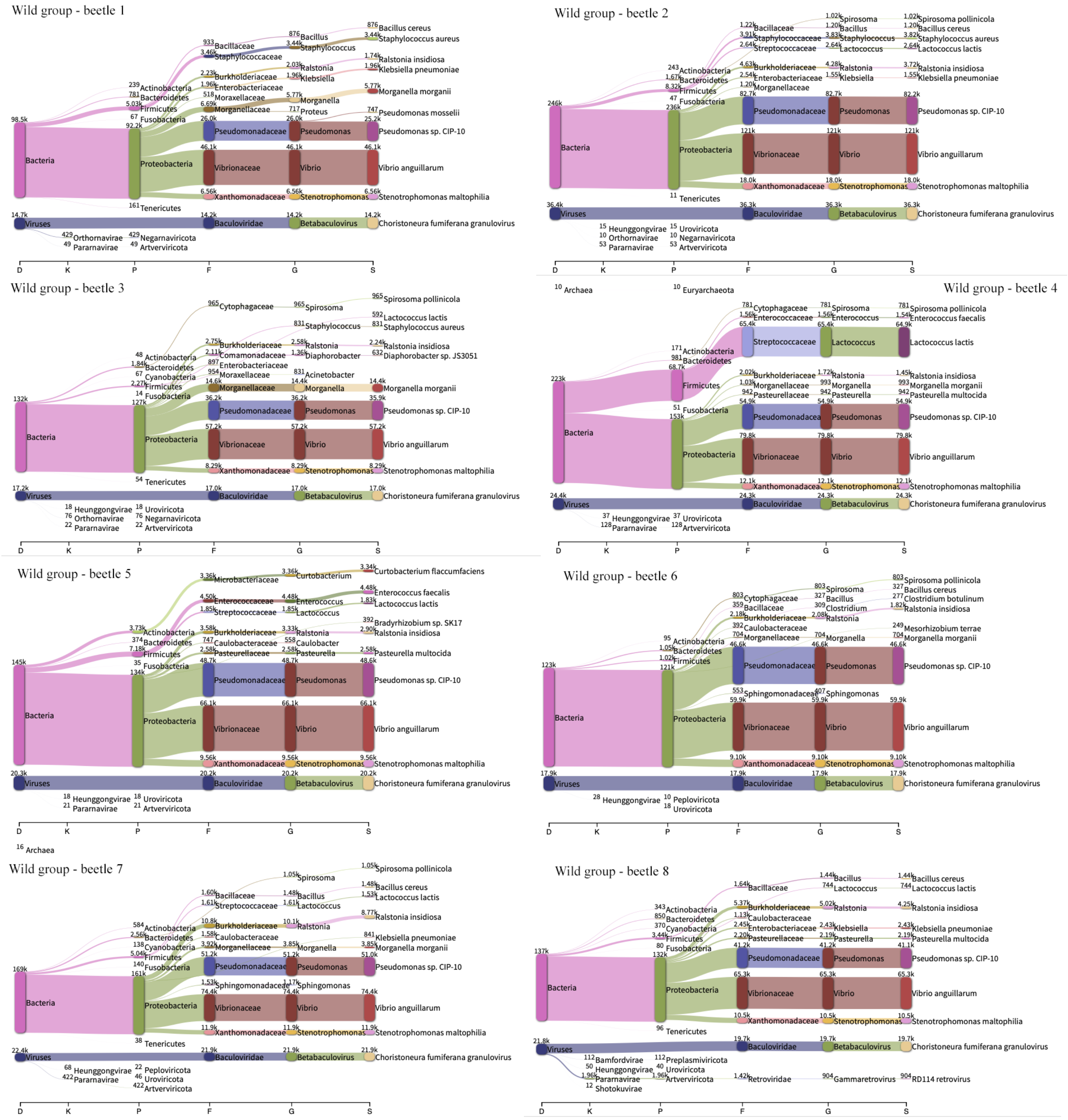

Figure S4 Pavian output for hierarchical visualization in the Wild group

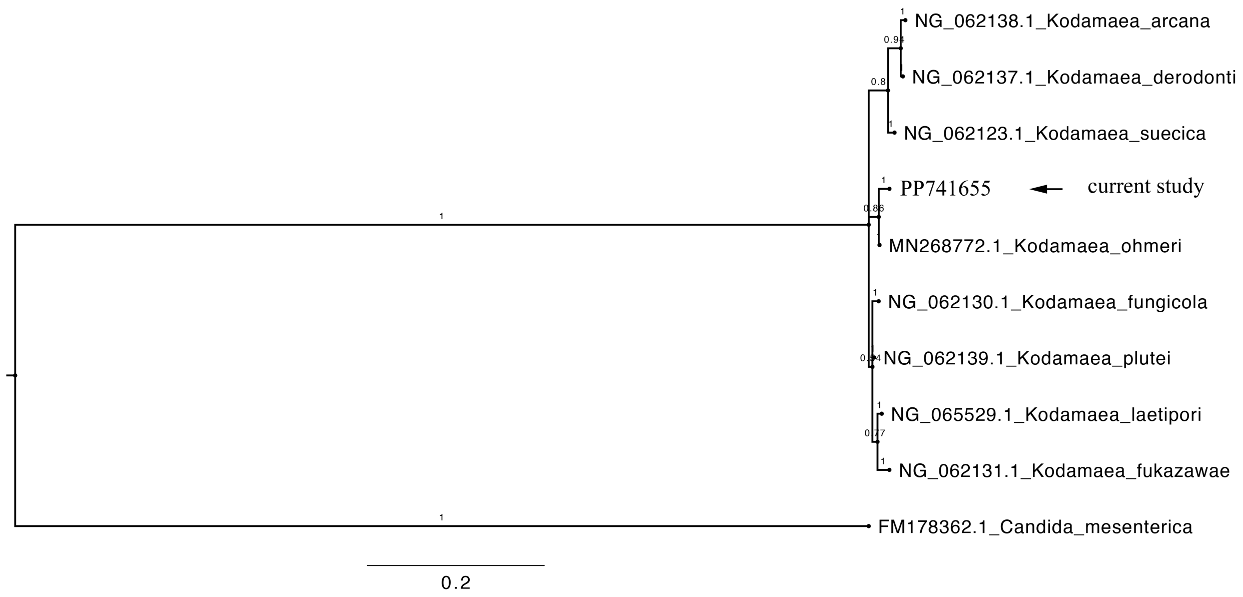

Figure S5 Phylogenetic tree of PCR sequence and *Kodamaea* species. The sequences were aligned with MUSCLE with default parameters and the tree was built using MrBayes with nchains=4, ngen=3000000, rates=invgamma, nst=6, resulting a deviation of 0.002. The tree was viewed using FigTree. The arrow indicates the PCR sequence in this study, which clustered with *K. ohmeri*, a symbiont of SHB. The legend indicates the branch length at the bottom.
